# Supplementary material for: What Is Wrong with Hyaluronic Acid Chemistry? A 15N/13C Solid-State NMR Re-Evaluation of Its Dopamine Conjugates
Source: Polymers (Basel). 2023 Jun 26;15(13):2825. doi: 10.3390/polym15132825 (PMC10347142; doi:10.3390/polym15132825)
Supplement: Supplementary file 1 [file polymers-15-02825-s001.zip › polymers-2441396-supplementary.pdf]

Supplementary Materials

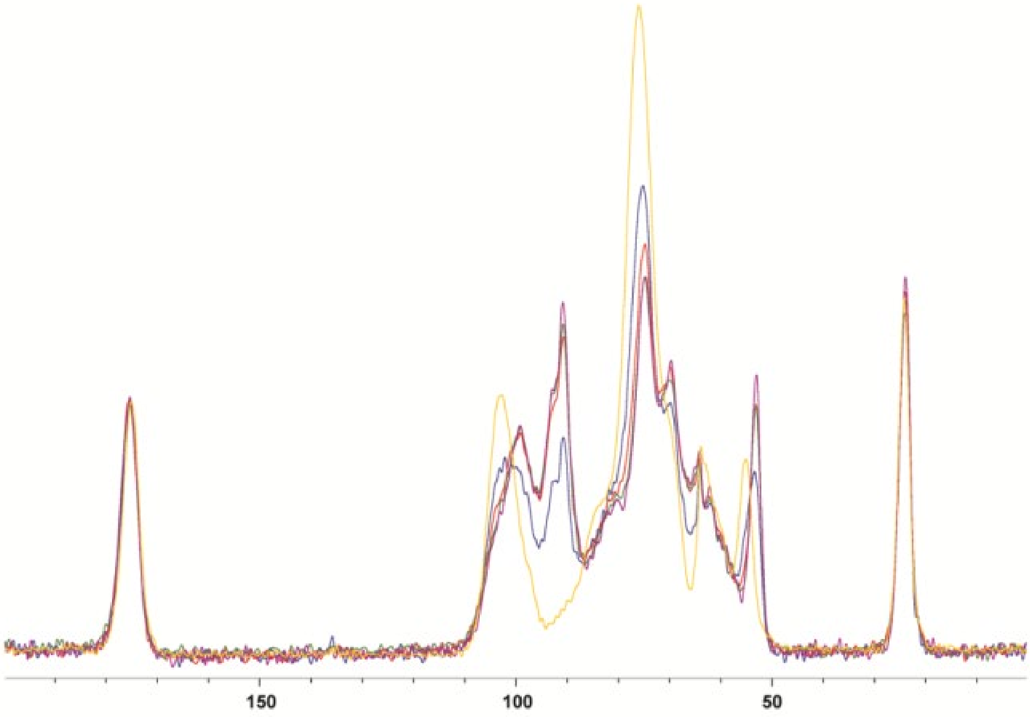

Figure S1. Comparison between the ^{13}C ss-NMR spectra recorded by the CP-MAS sequence on hyaluronic acid (in yellow) and $NaIO_4$-oxidized hyaloronic acid prepared as described in the Experimental section at the following $NaIO_4$/HA molar ratios: 1/1 (blue), 1/2 (red), 1/3 (green) and 1/4 (magenta). The most prominent new features in the oxidized hyaluronic acid are the occurrence of the extra-peak at 91–93 ppm, assigned to hemiacetals, and a slight shift of the ss-NMR peak at 103 ppm (which contains contributions from carbon sites next to the hemiacetals).

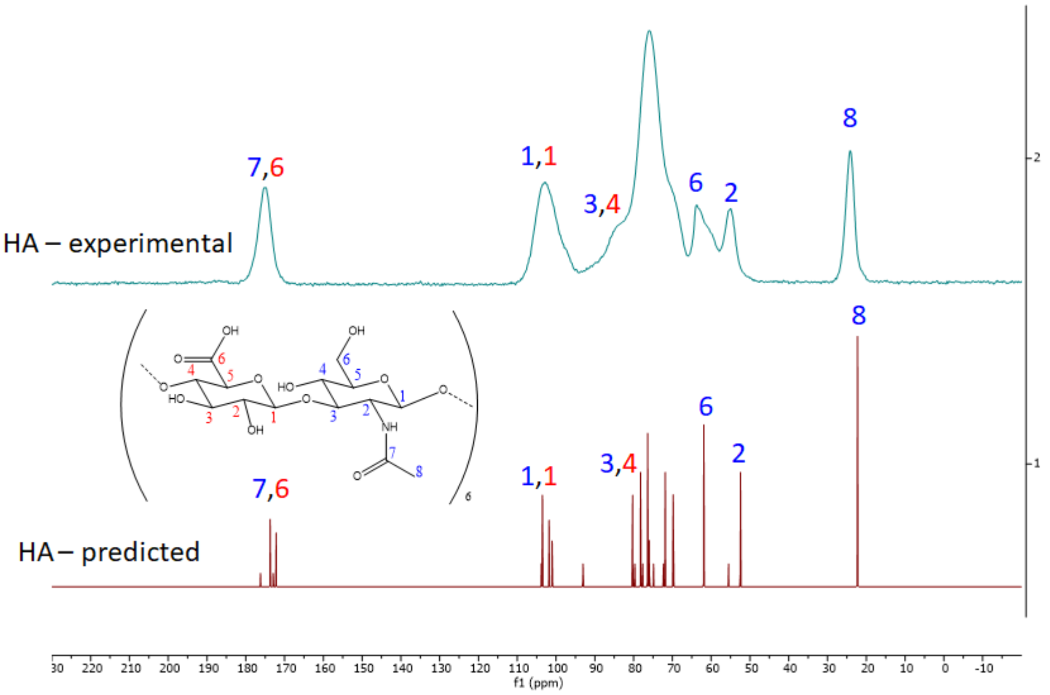

Figure S2. Comparison between the experimental ^{13}C ss-NMR spectrum and the predicted spectrum, as calculated by MestReNova, of hyaluronic acid.

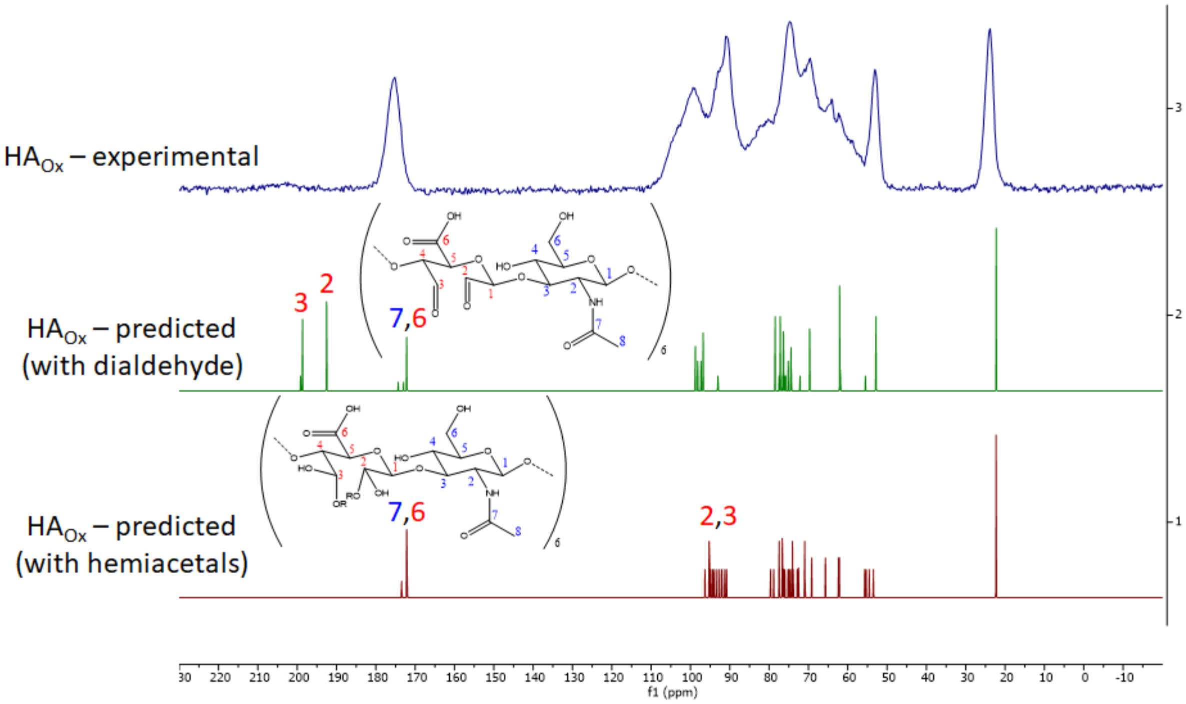

Figure S3. Comparison between the experimental ^{13}C ss-NMR spectrum and two MestReNova predicted spectra of $NaIO_4$ oxidized hyaluronic acid, using either dialdehyde or hemiacetals in their chemical structures.
